# Reduced survival of total knee arthroplasty after previous unicompartmental knee arthroplasty compared with previous high tibial osteotomy: a propensity-score weighted mid-term cohort study based on 2,133 observations from the Danish Knee Arthroplasty Registry

**DOI:** 10.1080/17453674.2019.1709711

**Published:** 2020-01-13

**Authors:** Anders El-Galaly, Poul T Nielsen, Andreas Kappel, Steen L Jensen

**Affiliations:** a Orthopaedic Research Unit, Aalborg University Hospital, Aalborg;; b Department of Clinical Medicine, Aalborg University, Aalborg, Denmark

## Abstract

Background and purpose — Both medial unicompartmental knee arthroplasties (UKA) and high tibial osteotomies (HTO) are reliable treatments for isolated medial knee osteoarthritis. However, both may with time need conversion to a total knee arthroplasty (TKA). We conducted the largest nationwide registry comparison of the survival of TKA following UKA with TKA following HTO.

Patients and methods — From the Danish Knee Arthroplasty Registry, aseptic conversions to TKA from UKA and TKA converted from HTO within the period of 1997–2018 were retrieved. The Kaplan–Meier method and the Cox proportional hazards regression were used to estimate the survival and hazard ratio (HR) for revision, considering confounding by indication utilizing propensity-score based inverse probability of treatment weighting (PS-IPTW).

Results — PS-IPTW yielded a well-balanced pseudo-cohort (standard mean difference (SMD) < 0.1 for all covariates, except implant supplementation) of 963.8 TKAs following UKA and 1139.1 TKAs following HTO. The survival of TKA following UKA was significantly less than that of TKA following HTO with a 5-year estimated survival of 0.88 (95% confidence interval (CI) 0.85–0.90) and 0.94 (CI 0.93–0.96), respectively. The differences in survival corresponded to an implant-supplementation adjusted HR of 2.7 (CI 2.4–3.1) for TKA following UKA compared with TKA following HTO.

Interpretation — Previous UKA more than doubled the revision risk of a subsequent TKA compared with previous HTO. This potential risk should be considered in the shared treatment decision of patients who are candidates for both UKA and HTO.

In isolated osteoarthritis of the medial knee compartment, both medial unicompartmental knee arthroplasties (UKA) and high tibial osteotomies (HTO) are solutions with reliable clinical outcomes (Cao et al. 2018). The survival of UKA is secondary to that of total knee arthroplasties (TKA) with a recent meta-analysis reporting 15-year survival of 76% and 93%, respectively (Evans et al. 2019). The long-term survival of HTO seems inferior to both UKA and TKA with a declining survival from 75% at 10 years to 55% at 15 years (van Wulfften Palthe et al. 2018). When UKA or HTO fail, conversion to TKA is a common solution (Lee et al. 2019). Nationwide registry studies have investigated the survival of either TKA following UKA or TKA following HTO compared with primary or revision TKA. They have reported an increased risk of revision in TKA following UKA (Robertsson and W-Dahl 2015, Leta et al. 2016, Lewis et al. 2018, El-Galaly et al. 2019) while no consensus regarding the influence of HTO on the survival of a subsequent TKA has been reached (Niinimäki et al. 2014, Badawy et al. 2015, Robertsson and W-Dahl 2015, El-Galaly et al. 2018). However, a direct comparison of the survival estimates from these studies is prone to confounding by indication due to a range of unadjusted baseline characteristics associated with the survival of TKA, such as implant constraints and hospital volume of arthroplasty surgeries (Jasper et al. 2016). This concern is further encouraged by a recent single-center study reporting similar short-term survival of TKA following UKA and TKA following HTO (Lim et al. 2017). Based on the Danish Knee Arthroplasty Registry (DKR), our study compares the survival of TKA converted from UKA with TKA converted from HTO with consideration for confounding by indication utilizing propensity-score based inverse probability of treatment weighting (PS-IPTW) (Inacio et al. 2015).  

## Patients and methods

### Data source

Since 1997, the DKR has prospectively collected information on Danish knee arthroplasties through standardized forms completed by surgeons. Since 2007, the registration of arthroplasties has been mandatory for all hospitals in Denmark leading to a registry completeness above 90% for primary arthroplasties and 80% for revision arthroplasties (Danish Knee Arthroplasty Registry 2019). The DKR is reported suitable for epidemiological studies and is crosslinked with the Danish Civil Registration System (DCRS) which contains vital and emigration status for all Danish citizens (Pedersen et al. 2012, Schmidt et al. 2014). Mandatory registration and linkage to the DCRS enable complete follow-up in a population-based cohort (Schmidt et al. 2019).

### Study cohort

**Figure 1.**
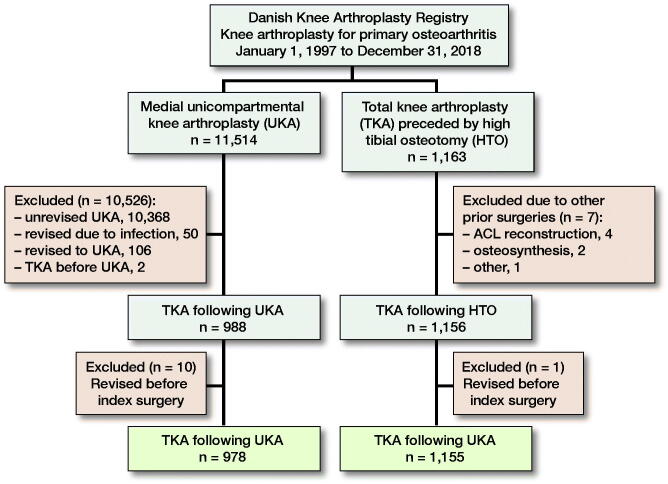
Flowchart depicting the formation of the study cohort.

In the registry, each patient is identified by a unique code, and the side of surgery is denoted. Therefore, each knee can be considered a unique observation, which has been reported to provide unbiased results in large arthroplasty studies (Robertsson and Ranstam 2003). All UKAs indicated by osteoarthritis in knees without prior surgery from January 1, 1997 until December 31, 2018 were retrieved. We identified later revisions conducted on the same knee and excluded revisions to UKA and conversions from UKA to TKA due to infections (Figure 1). In the same timeframe, we retrieved all TKAs indicated by osteoarthritis in knees previously treated with HTO. The validity of the registration of a previous HTO was evaluated in a recent study and confirmed in 96% of the cases (El-Galaly et al. 2018). However, HTO is not divided in open- and closed-wedge osteotomies, and thus the methods are considered as one in the DKR.

### Covariates

At the time of TKA surgery, several patients and surgical characteristics are registered in the DKR (Table 1). Comorbidity is depicted by Charnley class sorted into class A (unilateral arthritis), class B1 (bilateral arthritis), class B2 (arthroplasty in the opposite knee), and class C (other condition affecting walking capacity) (Bjorgul et al. 2010). Knee function at the time of surgery is registered using American Knee Society Score (KKS) divided into clinical and functional sub-scores, both ranging from 0 to 100 (Insall et al. 1989). Level of implant constraint is divided into cruciate retaining (CR), posterior stabilized (PS), constraint condylar (CCR), and hinged. Perioperative supplementation (stems, augments, or cones) and perioperative complications (e.g. fractures, rupture of the patellar tendon or ligament injuries) are registered. We defined the hospital volume of arthroplasties as the mean annual volume during the study period and divided it into 4 groups (< 100, 100–249, 250–449, > 449). Year of surgery was classified into 2 periods (1997–2007 and 2008–2018). For TKAs following UKAs, we retrieved type of UKA bearings and indications of conversion to TKA.

**Table 1.** Baseline characteristics for original cohort and the PS-IPTW cohort at the time of conversion to TKA

	Original cohort			PS-IPTW cohort		
Patient characteristics	TKA following UKA	TKA following HTO	SMD	TKA following UKA	TKA following HTO	SMD
Observations	978	1,155		963.8	1139.1	
Male sex, n (%)	324 (33)	657 (57)	0.24	419.3 (44)	529.4 (47)	0.03
Mean age (range)	66 (34–95)	63 (32–90)	0.35	64 (34–95)	64 (32–90)	0.06
Mean weight, kg (range)	82 (30–183)	84 (30–200)	0.04	89 (30–183)	88 (30–200)	0.05
Charnley class, n (%)			0.41			0.06
A	531 (54)	449 (39)		450.0 (47)	523.0 (46)	
B1	181 (18)	414 (36)		254.0 (26)	327.6 (29)	
B2	211 (22)	244 (21)		215.4 (22)	239.5 (21)	
C	55 (6)	48 (4)		44.3 (5)	49.0 (4)	
Knee Society Clinical score			0.22			0.02
mean (range)	41 (0–99)	35 (0–99)		35 (0–99)	36 (0–99)	
Knee Society Functional score			0.28			0.06
mean (range)	45 (0–100)	52 (0–100)		50 (0–100)	51 (0–100)	
Surgical characteristics, n (%)						
Level of constraint			0.62			0.06
Cruciate retaining	574 (59)	977 (85)		686.6 (71)	840.9 (74)	
Posterior stabilized	241 (25)	130 (11)		183.9 (19)	195.8 (17)	
Constrained condylar	161 (16)	44 (4)		90.9 (10)	99.3 (9)	
Hinged	2 (< 1)	4 (< 1)		2.3 (< 1)	3.1 (< 1)	
Fixation			0.49			0.07
Cemented	895 (91)	848 (73)		806.2 (84)	922.7 (81)	
Hybrid	68 (7)	228 (20)		115.7 (12)	160.0 (14)	
Uncemented	15 (2)	79 (7)		41.9 (4)	56.4 (5)	
Patella resurfacing	904 (78)	862 (88)	0.10	822.4 (85)	948.5 (83)	0.02
Supplementation			0.80			0.58
Stem	271 (28)	27 (2)		216.5 (23)	51.6 (5)	
Augment	75 (8)	2 (< 1)		50.2 (5)	2.9 (< 1)	
Cone	59 (6)	1 (< 1)		31.5 (3)	6.1 (< 1)	
Annual arthroplasty volume			0.35			0.09
< 100	80 (8)	176 (15)		148.9 (15)	143.8 (13)	
100–249	218 (22)	321 (28)		217.1 (23)	277.9 (24)	
250–449	373 (38)	276 (24)		300.4 (31)	359.0 (31)	
> 449	307 (32)	382 (33)		297.4 (31)	358.4 (32)	
Period of surgery			0.38			0.01
1997–2007	144 (15)	604 (52)		333.3 (35)	405.9 (36)	
2008–2018	834 (85)	551 (48)		630.5 (65)	733.2 (64)	

SMD: Standardized mean difference.

### Outcome

The outcome was TKA revision of any indication with revision defined in accordance with the DKR as removal, exchange, or addition of an implant. The indications for TKA revision have recently been thoroughly evaluated in both groups, and thus are not presented in this study (El-Galaly et al. 2018, 2019).

### Missing values

Missing values existed in height (n = 1,214), weight (n = 67), KSS clinical sub-score (n = 55), KSS functional sub-score (n = 39), Charnley class (n = 12), fixation (n = 11), duration of surgery (n = 8), and patella replacement (n = 3). Missing values in height were deemed too high for meaningful imputation and discarded. The remaining missing values were estimated by multiple imputation with chain equation (MICE), generating 5 datasets under the assumption of missing at random (Azur et al. 2011).

### Statistics

#### PS-IPTW

This study is subjected to confounding due to the non-random assignment of prior UKA or HTO. Therefore, PS-IPTW was utilized to account for confounding by indication. PS were estimated with logistic regression and applied by IPTW with stabilized weights aiming to estimate the average effect of treatment (Austin 2014). Based on the considerations depicted in the directed acyclic graph (Williams et al. 2018) (Figure 2, see Supplementary data), the following covariates were included in the model: sex, age (quantiles), weight (quantiles), KSSs, Charnley class, level of constraint, patella resurfacing, fixation, hospitals annual arthroplasty volume, and period of surgery. Implant supplementation was rare in TKA following HTO and therefore omitted from the PS estimation to avoid overweighting rare observations. The balance of the baseline characteristics was evaluated graphically and by standardized mean differences (SMD) with an SMD of 0 indicating perfect balance and SMD < 0.1 deemed an acceptable balance between the groups (Austin 2009).

### Survival analyses

The Kaplan–Meier method was used to estimate the survival with revision as the primary endpoint. Unrevised knees were censored by death, emigration, or end of study period at December 31, 2018. The risk of revision was estimated by Cox regression with robust variance estimator to account for dependencies in the PS-IPTW cohort. The assumption of proportional hazards was evaluated by Schoenfeld’s plots and Schoenfeld’s residual test. Implant supplementation was included as covariate in the Cox regression to account for remaining imbalance following the PS-IPTW.

### E-value

The robustness of the estimated hazard ratios (HR) was evaluated by calculating their E-values, which estimates the magnitude of association unmeasured confounders must have with both the exposure and outcome to negate the observed HRs (Van Der Weele and Ding 2017).

### Significance

Means are presented with absolute range, medians with interquartile range (IQR), and SMD are calculated to assess balance between the groups. Estimates from the imputed datasets were combined by Rubin’s rule (White et al. 2011), and all estimates are presented with 95% confidence interval (CI) to address their significance (Ranstam 2019).

### Statistical programs

Data were sorted in STATA 15 (StataCorp, College Station, TX, USA) and all analyses were conducted in R© Version 3.5.1 (R Foundation for Statistical Computing, Vienna, Austria).

### Ethics, funding, and potential conflict of interests

The study was approved by the Danish Data Protection Agency (entry number: 2008-58-0028) and financed by the Orthopaedic Research Unit at Aalborg University Hospital. No conflict of interest is present among the authors.

## Results

### Original cohort

#### Characteristics

The baseline covariates differed in sex, age, Charnley class, KKSs, level of constraint, patella resurfacing, fixation, implant supplementation, annual arthroplasty volume, and period of surgery as depicted in Table 1. The median duration of surgery in TKA following UKA was 90 minutes (IQR 75–115) compared with 80 minutes (IQR 65–100) in TKA following HTO (SMD = 0.35). In 12 of the TKAs following UKAs perioperative complications (8 fractures, 4 other) were registered compared with 18 registered complications (5 fractures, 5 ligament/tendon rupture, 8 other) in TKAs following HTOs (SMD = 0.11).

#### Survival

Of the 978 TKAs following UKAs, 121 (12%) were revised within the study period and 93 (10%) were censored due to either death or emigration. In comparison, 101 (9%) and 234 (20%) of the 1,155 TKAs following HTOs were revised or censored, respectively. The median follow-up in TKA following UKA was 4.7 years (IQR 1.9–7.7) compared with 9.3 years (IQR 5.0–13) for TKA following HTO (SMD = 0.87). The 1st, 5th, and 10th year survival estimates were 0.97 (CI 0.96–0.98), 0.88 (CI 0.86–0.91), and 0. 82 (CI 0.78–0.85) for TKA following UKA compared with 0.98 (CI 0.97–0.99), 0.95 (CI 0.93–0.96), and 0.92 (CI 0.90–0.94) for TKA following HTO, which corresponds to an HR of 2.3 (CI 2.1–2.6) associated with TKA following UKA (Table 3).

**Table 3.** Survival estimates, hazard ratios (HR), and E-value for the original cohort and PS-IPTW cohort

	n	Follow-up median (IQR)	Revision n (%)	1-year (CI)	Survival estimates 5-year (CI)	10-year (CI)	Hazard ratio estimates (CI)	E-value estimates (lower CI)
Original cohort								
TKA following UKA	978	4.7 (1.9–7.7)	121 (12)	0.97 (0.96–0.98)	0.88 (0.86–0.91)	0.82 (0.78–0.85)	2.3 (2.1–2.6)	4.1 (3.5)
TKA following HTO	1155	9.3 (5.0–13.4)	101 (9)	0.98 (0.97–0.99)	0.95 (0.93–0.96)	0.92 (0.90–0.94)	Ref.	Ref.
PS-IPTW cohort								
TKA following UKA	963.8	5.5 (2.1–9.3)	169.1 (17)	0.96 (0.95–0.97)	0.88 (0.85–0.90)	0.75 (0.71–0.79)	2.7 **^a^**(2.4–3.1)	4.9 (4.3)
TKA following HTO	1139.1	7.8 (4.2–11.2)	89.4 (8)	0.98 (0.97–0.99)	0.94 (0.93–0.96)	0.92 (0.90–0.94)	Ref.	Ref.

a adjusted for differences in implant supplementation.

### PS-IPTW cohort

#### Characteristics

Following PS-IPTW, all covariates included in the estimation of the PS were well balanced between TKAs following UKAs and TKAs following HTOs (Table 1 and Figure 3, see Supplementary data). However, the difference in implant supplementation was still unbalanced following PS-IPTW (SMD = 0.58). Table 2 depicts the distribution of indication of UKA conversion and type of UKA-bearing in TKA following UKA, which was clinically comparable before and after PS-IPTW. The imbalance in duration of surgery was unchanged by PS-IPTW with a median duration of 90 minutes (IQR 75–120) in TKA following UKA and 80 minutes in TKA following HTO (IQR 66–100) (SMD = 0.35). PS-IPTW did not balance the difference in perioperative complications with registered complications in 7.7 of the TKAs following UKAs and 24.1 in TKAs following HTOs (SMD = 0.13).

**Table 2.** Baseline characteristics for original cohort and PS-IPTW cohort at the time of conversion to TKA. Values are counts/weighted counts (%)

	Prior UKA surgery	
	Original cohort	PS-IPTW cohort
Indications for conversion		
Aseptic loosening	271 (28)	285.4 (29)
Unexplained pain	262 (27)	274.7 (29)
Progression of arthritis	243 (25)	207.2 (22)
Instability	77 (8)	69.3 (7)
Other	65 (6)	68.7 (7)
Unknown	44 (4)	45.4 (5)
Wear	16 (2)	13.0 (1)
Bearing		
Mobile	823 (84)	759.0 (79)
Fixed	155 (16)	204.8 (21)

#### Survival

**Figure 4.**
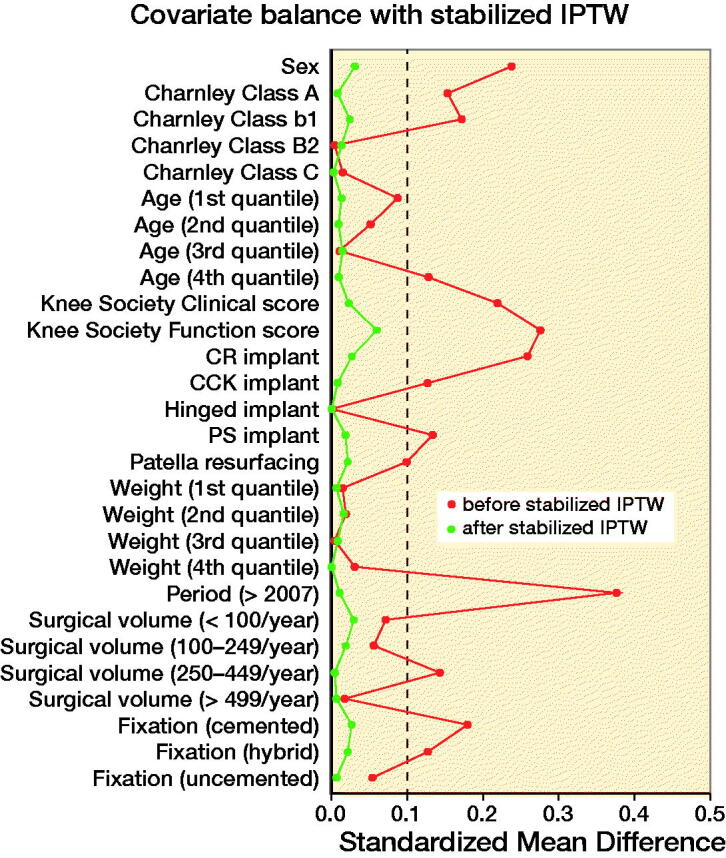
Kaplan–Meier survival estimates for the PS-IPTW cohort with confidence interval and weighted knees at risk.

Of the 963.8 TKAs following UKAs, 169.1 (17%) were revised within the study period and 117.5 (12%) were censored due to death or emigration. Similarly, 89.4 (8%) and 187.8 (16%) of the 1139.1 TKAs following HTOs were revised or censored, respectively. This corresponded to a significantly inferior survival of TKA following UKA compared with TKA following HTO (Figure 4 and Table 3), with an implant-supplementation adjusted HR of 2.7 (CI 2.4–3.1) associated with TKA following UKA (Table 3). 

## Discussion

Our study showed that in a cohort from the Danish Knee Arthroplasty Registry, with well-balanced baseline covariates, the survival of TKA following UKA was lower than the survival of TKA following HTO. More specifically, the risk of revision more than doubled when TKA was preceded by UKA compared with HTO.

During recent years, UKA has gained popularity while the use of HTO has decreased, indicating a trend towards treating patients with UKA instead of HTO (Niinimäki et al. 2012, Henkel et al. 2019). While both UKAs and HTOs relieve pain, the procedures differ. UKAs replace the diseased compartment with an implant and thus preserve the mechanical axis of the knee. In contrast, HTOs shift the mechanical axis laterally, unloading the diseased compartment while increasing the load on the lateral compartment. Therefore, a subsequent conversion from HTO to TKA due to progression of osteoarthritis might be expected, whereas progression of arthritis or implant failure are considered an adverse event in UKA surgery. To our knowledge, this study constitutes the largest direct comparison of TKA following UKA with TKA following HTO based on a nationwide registry (Pearse et al. 2012) and expands upon a range of recent nationwide registry studies comparing the survival of either TKA following UKA or TKA following HTO with primary TKA (Niinimäki et al. 2014, Badawy et al. 2015, Robertsson and W-Dahl 2015, Leta et al. 2016, El-Galaly et al. 2018, 2019, Lewis et al. 2018). Due to inconsistent adjustment for confounding, a direct comparison of the results in the current literature might be affected by residual confounding, which could result in acceptance of a false causal relationship (Kyriacou and Lewis 2016). Our study compliments current literature by directly comparing TKA following UKA with TKA following HTO while expanding the statistical adjustment for confounding using PS-IPTW. The PS is the probability of an observation receiving a treatment given a set of baseline covariates and, thus, dependence on the PS creates balance in the included covariates between the groups. Dependence on the PS can be achieved by matching, weighting, adjusting, or stratifying (Austin 2011). We used IPTW and, thus, weighted the observation based on their inverse probability of treatment (i.e., PS) to create a pseudo-cohort with comparable baseline characteristics between the groups. As depicted in Figure 3 (see Supplementary data), this approach eliminated imbalances in a range of baseline covariates, and thus diminished the influence of the confounders presented in Figure 2 (see Supplementary data) except implant supplementation, which was including in the Cox regression. In this pseudo-cohort, TKA following UKA was associated with a 2.7-fold increase in the risk of revision compared with TKA following HTO.

### Limitations

The study has some limitations. First, nationwide registries are prone to misclassifications. However, as the data are collected prospectively by the surgeon on a standardized form, the misclassifications are assumed to be non-differential and thus bias the results towards no difference between the groups. Second, even though the PS-IPTW successfully balances a wide range of covariates, residual confounding is unavoidable in non-randomized studies. We calculated the E-value for the presented HRs to elucidate which magnitude unmeasured confounders must have to negate the presented HRs (Table 3). The E-value indicated that unmeasured confounders must be associated with both TKA following UKA (exposure) and subsequent revisions (outcome) by a ratio of at least 4.3 (lower CI) to move the HR’s CI to include 1. In comparison, diabetes has recently been associated with a risk ratio of revision at 1.3 (CI 1.02–1.6) in a large retrospective study of both TKAs and total hip arthroplasties (Maradit Kremers et al. 2017). Therefore, the presented HRs seemed robust for residual confounding. Third, the completeness in the DKR has increased from 69% of primary arthroplasties in 1997 to above 91% since 2008, with a similar evolution in revision arthroplasties with a completeness from 54% in 1997 to above 87% since 2008 (Danish Knee Arthroplasty Registry 2019). The overall completeness of TKA following HTO might be less than the overall completeness of TKA following UKA, as more HTOs were converted before 2008. This imbalance might have overestimated the risk of revision associated with TKA following UKA compared with TKA following HTO. We included the period of surgery in the PS estimation to contain the bias induced by the difference in completeness.

### Conclusion

In this propensity-score weighted cohort study, TKA following UKA was associated with a more than 2-fold increased risk of revision compared with TKA following HTO. This potential risk emphasized that UKA should be considered a definitive treatment in line with TKA rather than a temporary treatment to postpone TKA.

### Supplementary data

Figures 2 and 3 are available as supplementary data in the online version of this article, http://dx.doi.org/10.1080/17453674.2019.1709711

The authors thank the Danish knee surgeons for a thorough registration of their procedures, the steering committee of the Danish Knee Arthroplasty Registry for their goodwill in relation to data acquisition, and Angélica Meleñdez-Muñoz for her linguistic contribution to the manuscript.

All authors contributed to the study design. AEG received and analyzed the data with supervision from all authors. AEG wrote the initial draft, which was revised and accepted by all authors.

*Acta* thanks Leif Ryd and Annette W-Dahl for help with peer review of this study.

## Supplementary Material

Supplemental Material
