# Supplementary material for: Reduced survival of total knee arthroplasty after previous unicompartmental knee arthroplasty compared with previous high tibial osteotomy: a propensity-score weighted mid-term cohort study based on 2,133 observations from the Danish Knee Arthroplasty Registry
Source: Acta Orthop. 2020 Jan 13;91(2):177–83. doi: 10.1080/17453674.2019.1709711 (PMC7144230; doi:10.1080/17453674.2019.1709711)
Supplement: Supplemental Material [file IORT_A_1709711_SM0274.pdf]

Supplementary data

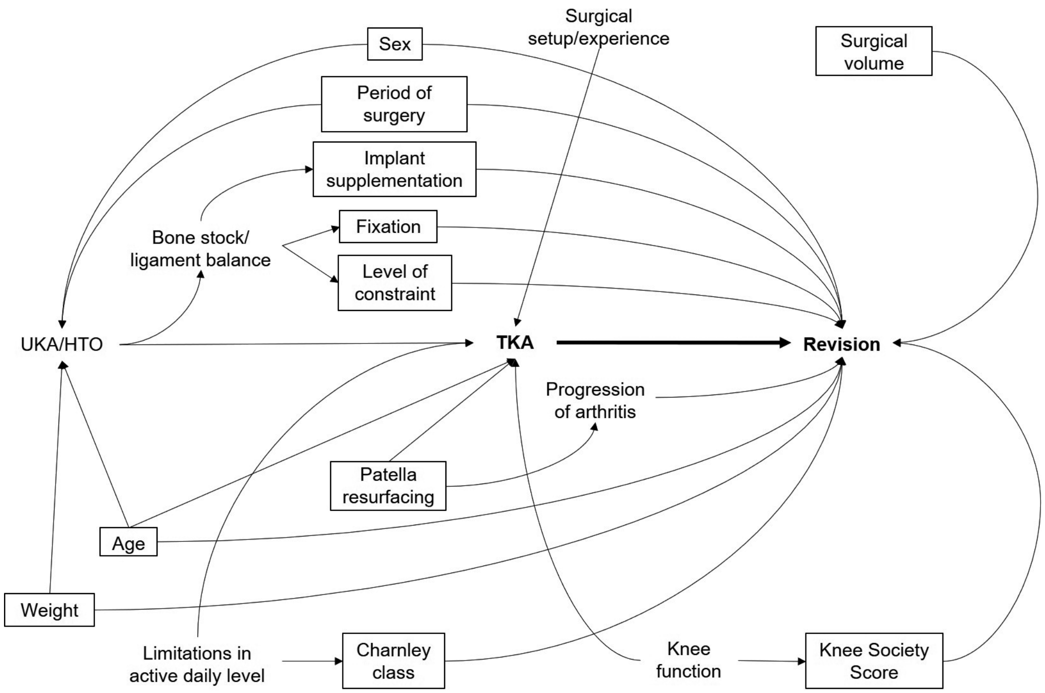

Figure 2. A priori consideration for the included confounders. The considerations are presented as a directed acyclic graph (DAG) with boxed covariates indicating a blocked pathway. A DAG is a graphical illustration of confounding consideration with arrows indication causality.

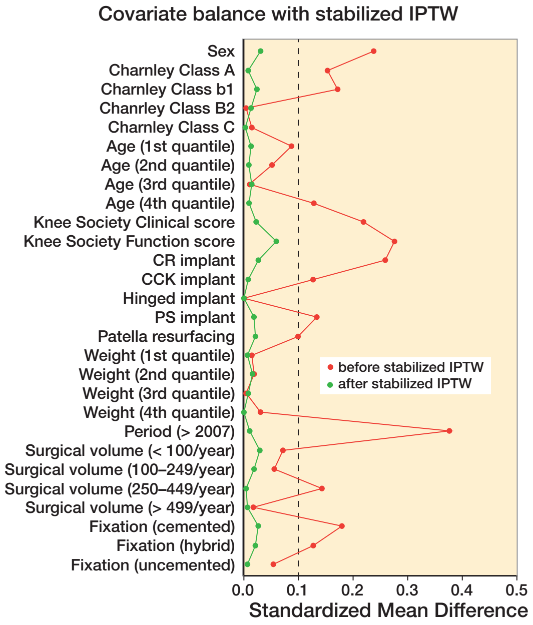

Figure 3. Covariate balance following stabilized inverse probability treatment weighting using propensity score (PS-IPTW).
